# Supplementary material for: A Versatile Clustered Regularly Interspaced Palindromic Repeats Toolbox to Study Neurological CaV3.2 Channelopathies by Promoter-Mediated Transcription Control
Source: Front Mol Neurosci. 2022 Jan 6;14:667143. doi: 10.3389/fnmol.2021.667143 (PMC8770422; doi:10.3389/fnmol.2021.667143)
Supplement: Supplementary file 1 [file Data_Sheet_1.pdf]

A versatile CRISPR toolbox to study neurological $Ca_V3.2$ channelopathies by promoter-mediated transcription control

Supplementary Material

Supplementary Table 1. Primers used for cloning

**Construct name**	**Primer sequence 5´-3´**
pAAV-U6-sgRNA	forward: ctgcggccgcacgcggagggcctatttcccatgattcc<br>reverse: aattcaatcgatgcgggtacctctagagccatttgt
pAAV-sgRNA1-*Cacna1h*	Forward: caccggggcgtcgttcctgggcca<br>Reverse: aaactggcccaggaacgacgcccc
pAAV-sgRNA2-*Cacna1h*	Forward: caccgagagacaaagacatcccgg<br>Reverse: aaacccgggatgtctttgtctctc
pAAV-sgRNA-*Lac*Z	Forward: caccgtgcgaatacgcccacgcgat<br>Reverse: aaacatcgcgtgggcgtattcgcac
pLenti-syn-dCas9-VPR	Forward: gtttggttaattaagagtgcaagtgggttttaggacc<br>Reverse: atagtcggtggcagcatcgatgcgatcgcatgc
pLenti-U6-sgRNA$_{(Cacna1h/LacZ)}$-Syn-dCas9-VPR	forward: gatccagtttggttagagggcctatttcccatgattcct<br>reverse: ccacttgcactcttactagagccatttgtctgcagaat
pLenti-syn-dCas9-KRAB-T2A-eGFP	forward: gatccagtttggttaattaagtagactgcagagggccctg<br>reverse: ccatggtggctctagagaattcaatcgatgcgatcgcatgcgc
pLenti-U6-sgRNA$_{(Cacna1h/LacZ)}$-Syn-dCas9-KRAB-T2A-eGFP	Forward: gatccagtttggttagagggcctatttcccatgattcct<br>reverse: tctgcagtctacttagcgcacgcgctaaaaacggact
pTRE-VPR	forward: gtttggttaattaagaaaccattattatcatgacattaacctataaaaataggcgt<br>reverse: atagtcggtggcagcggcgatctgacggttcactaaac
pTRE-KRAB-T2A-eGFP	forward:gatccagtttggttaattaacattattatcatgacattaacctataaaaataggcgtatcac<br>reverse: ccatggtggctctagaggcgatctgacggttcac
pAAV-hSyn-rtTA	forward: cgcatcgattgaattatgtctagactggacaagagcaaagt<br>reverse: gcttctgcaggtcgattacccggggagcatgtcaag

Supplementary Table 2. Primers used for Real time qPCR

**Gene**	**Primer sequence 5´-3´**
Synaptophysin	Forward: ttcaggactcaacacctcggt<br>Reverse: cacgaaccataggttgccaac
Cacna1h	Forward: atgtcatcaccatgtccatgga<br>Reverse: acgtagttgcagtacttaagggcc
Cacna1g	Forward: accctggcaagcttctctga<br>Reverse: gcggaggatgtacaccaggta
Cacna1i	Forward: tcatccgtatcatgcgtgttct<br>Reverse: gggcccgcattcctgt
Cacna1e	Forward: ggagtggatacccttcaatgagtc<br>Reverse: tctgttaccaccagagattgttgttc

Supplementary Figure 1.

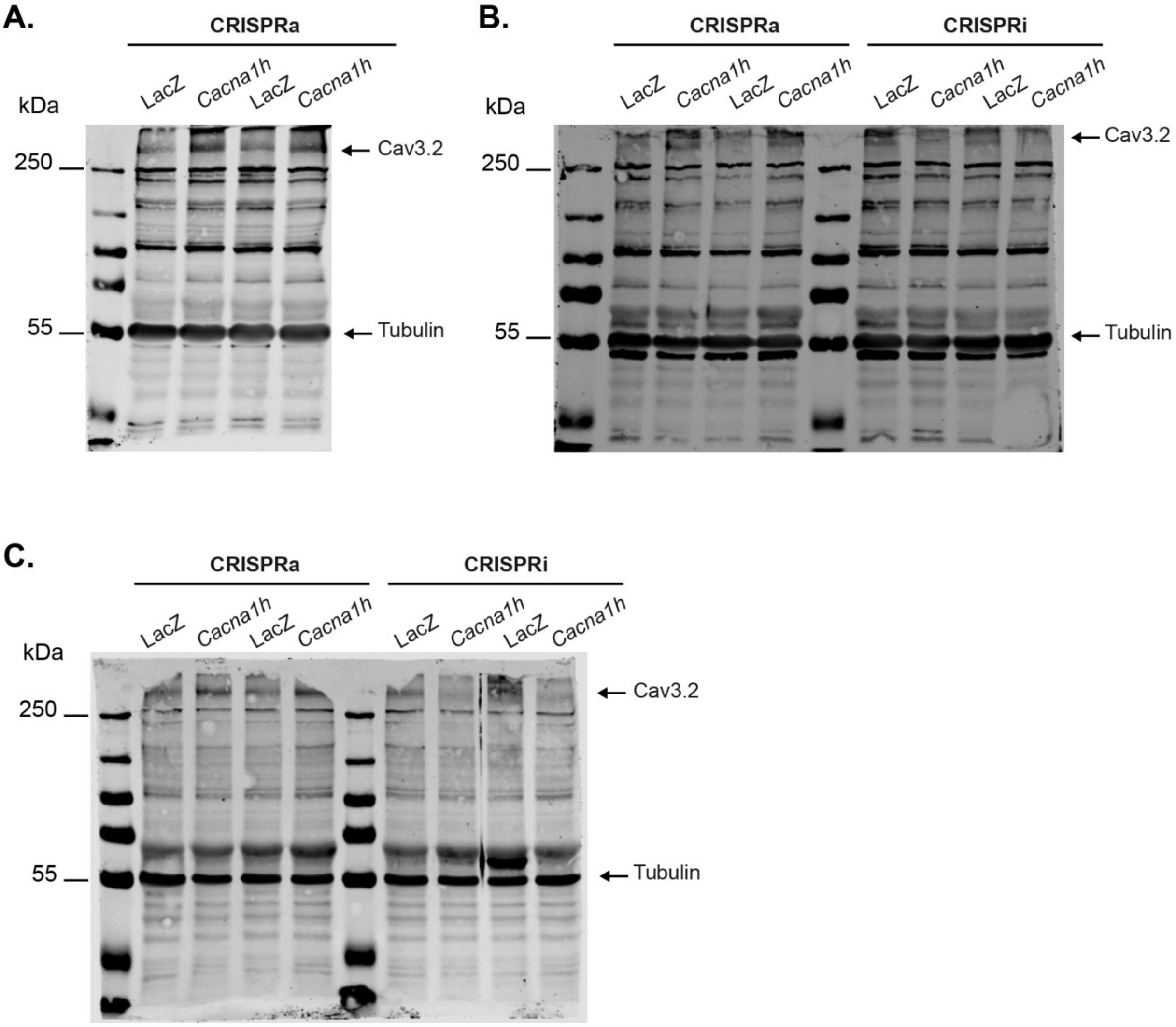

Supplementary Figure 1. Western blots for Cav3.2 in neuronal cultures transduced with CRISPRa/i-*Cacna1*h. Examples of immunoblots for Cav3.2 detected in neuronal cultures transduced with CRISPRa-Cacna1h/LacZ **(A)** and CRISPRa-*Cacna1h*/LacZ or CRISPRi-*Cacna1h*/LacZ **(B,C).** Each blot represents independent cultures. Tubulin was used as loading control.

Supplementary Figure 2.

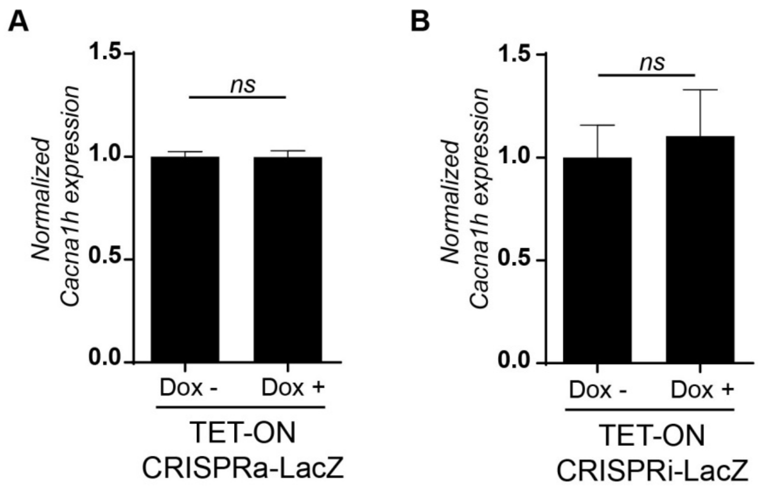

Supplementary Figure 2. TET-ON-CRISPRa/i system of *Lac*Z controls. (A) *Cacna1h* mRNA expression of cultured neurons after transduction with the TET-ON-CRISPRa-*Lac*Z system (pTRE-KRAB-T2A-eGFP, pAAV-hSyn-rtTA and pAAV-sgRNA-LacZ) in the presence or absence of doxycycline (1 µg/mL) (N=4, *t*-test). (**B**) *Cacna1h* mRNA expression of cultured neurons after transduction with the TET-ON-CRISPRi-LacZ system (pTRE-KRAB-T2A-eGFP, pAAV-hSyn-rtTA and pAAV-sgRNA-LacZ) in the presence or absence of doxycycline (1 µg/mL) (N=5, *t*-test).
